# *Paracoccidioides* Spp.: Virulence Factors and Immune-Evasion Strategies

**DOI:** 10.1155/2017/5313691

**Published:** 2017-05-02

**Authors:** Emma Camacho, Gustavo A. Niño-Vega

**Affiliations:** ^1^Department of Molecular Microbiology and Immunobiology, Johns Hopkins Bloomberg School of Public Health, Johns Hopkins University, Baltimore, MD, USA; ^2^Departamento de Biología, División de Ciencias Naturales y Exactas, Universidad de Guanajuato, Guanajuato, GTO, Mexico

## Abstract

*Paracoccidioides* spp. are dimorphic fungal pathogens responsible for one of the most relevant systemic mycoses in Latin America, paracoccidioidomycosis (PCM). Their exact ecological niche remains unknown; however, they have been isolated from soil samples and armadillos (*Dasypus novemcinctus*), which have been proposed as animal reservoir for these fungi. Human infection occurs by inhalation of conidia or mycelia fragments and is mostly associated with immunocompetent hosts inhabiting and/or working in endemic rural areas. In this review focusing on the pathogen perspective, we will discuss some of the microbial attributes and molecular mechanisms that enable *Paracoccidioides* spp. to tolerate, adapt, and ultimately avoid the host immune response, establishing infection.

## 1. Introduction

*Paracoccidioides* spp. are causative agents of paracoccidioidomycosis (PCM), a human systemic mycosis endemic to Latin America and one of the most prevalent deep mycoses of the region. PCM can go from an acute/subacute clinical type to a chronic progressive disease [1, 2]. Brazil accounts for over 80% of all reported cases, followed by Venezuela, Colombia, Ecuador, Bolivia, and Argentina [1].

To date, the genus *Paracoccidioides* have been reported as constituted by two species: *Paracoccidioides lutzii*, compose of a single monophyletic population so far found in Central West of Brazil and present in Ecuador [3–5], and *Paracoccidioides brasiliensis*, which comprises a complex of at least four cryptic species, namely, S1 (present in Southeast and Central West of Brazil, as well as Argentina), PS2 (found in Southeast Brazil and Venezuela), PS3 (restricted to Colombia), and PS4 (only found in Venezuela) [5–8]. Both species are thermodimorphic, growing as yeast-like multibudding cells, both in cultures at 37°C and in infected tissues, and as mycelium at temperatures of 20–23°C, which has been regarded as its environmental morphotype.

PCM is acquired by inhalation of conidia [9], and all *Paracoccidioides* species can cause both acute/subacute and chronic diseases although some differential clinical features have been observed in patients infected with either *P. lutzii* or the *P. brasiliensis* species complex [8, 10]. Indeed, infections reported in endemic areas of *P. lutzii* frequently present lymphatic-abdominal clinic manifestation, which are not reported in areas endemic for the *P. brasiliensis* species complex [10]. Also, sera recovered from patients infected with *P. lutzii* are not recognized by *P. brasiliensis* antigens and conversely [8, 11–13].

The interaction between *Paracoccidioides* spp. and its extracellular environment, either in their free-living stages or inside the host, has driven the molecular evolution of these fungi, particularly in the microbial components involved in virulence [14]. However, virulence is not an independent microbial property because it cannot be defined independently from a host. Virulence is the outcome of the interaction between a host and a microbe, whereas the host aims to effectively control the pathogen causing little or nontissue damage. Thusly, in the context of the “damage-response framework,” a virulence factor is a microbial component that can damage a susceptible host [15]. Furthermore, the successful microbial clearance after a microbial invasion into a mammalian host relies on the host cellular immunity, mediated by the cells of the innate and adaptive systems. An initial response involves dendritic cells' and macrophages' recognition and presentation of fungal antigens (e.g., chitin, *β*-glucans, and mannans), to T-lymphocytes (reviewed by [16]). Those fungal antigens are known as pathogen-associated molecular patterns (PAMPs), which are recognized by the cells of the innate immune system through receptors, namely, pattern recognition receptors (PRRs), such as Toll-like receptors (TLR), nucleotide-binding oligomerization domain- (NOD-) like proteins, and C-type lectin receptors (CLRs) (reviewed by [17]). During a later stage, an effective T-cell response must lead to the generation of Th1 cytokines, such as tumor necrosis factor (TNF-*α*) and interferon gamma (IFN-*γ*), resulting in a classic activation of macrophages to produce reactive oxygen species (ROS) and reactive nitrogen species (RNS) that kill fungi or inhibit their growth [16, 18].

Colonization and invasion of the host is based on a myriad of fungal components and strategies to bypass host defense mechanisms. Indeed, microbial attributes that confer *Paracoccidioides* spp. the potential to become pathogens are intimately related to escape strategies to avoid clearance and bypass host defense mechanisms. Identification of genes related to fungal virulence factors has occurred mainly using molecular tools to genetically manipulate these organisms. Functional analyses in the genus *Paracoccidioides* are still hindered by the highly complex task of achieving viable and stable mutants. In this review, we discuss some of the tools and strategies developed by *Paracoccidioides* spp. to efficiently evade/manipulate the host immune response, occasionally based on studies performed in other endemic dimorphic fungi (*Blastomyces dermatitidis*, *Histoplasma capsulatum*), where there is deeper understanding of the molecular mechanisms associated with key microbial components.

## 2. Adaptation

### 2.1. Morphogenesis

In *Paracoccidioides* spp., once conidia or hyphal fragments are inhaled into the lung alveoli, the morphological switch to multibudding yeast cells is a requirement for the disease to be established [19]. Therefore, the mechanisms involved in this morphological change are potential targets for the development of antifungal drugs against these dimorphic fungi. One of those mechanisms studied in *Paracoccidioides* spp. is the synthesis of polyamines, a metabolic process that has been related to the dimorphic change of some fungi [20]. These are micromolecules required for cellular growth and differentiation in eukaryotic systems and originated by the decarboxylation of ornithine by ornithine decarboxylase (ODC), which gives rise to putrescine, the first polyamine in the metabolic pathway. In *P. brasiliensis*, high levels of ODC activity are induced at the onset of the budding process during the yeast growth and during the mycelium-to-yeast transition in vitro [21, 22]. Also, the dimorphic transition can be repressed by the addition of the ODC inhibitor 1,4-diamino-2-butanone (DAB) [22].

In other fungi, at least three signaling pathways that induce dimorphic switching and yeast growth at 37°C have been identified: (a) the two-component signaling, (b) heterotrimeric G protein and Ras signaling, and (c) calcium signaling (reviewed by [23]). The two-component signaling system is regulated through DRK1 (dimorphism-regulating histidine kinase 1). Reports in *B*. *dermatitidis* and *H*. *capsulatum* showed that *DRK1* mutants are avirulent in a murine model of infection. These mutant strains fail to convert to the pathogenic yeast form and grow as mycelia at 37°C [24]. In *Paracoccidioides* spp., an ortholog *DKR1* is highly expressed in the virulence phase and is fundamental in the mycelia-to-yeast transition [25, 26]. Ras GTPases are shown to control multiple processes including cAMP signaling, morphogenesis, differentiation, cell cycle progression, and fungal pathogenic gene expression [23]. Evidences that a heterotrimeric G protein and the Ras signaling pathway influence dimorphic switching in *Paracoccidioides* spp. were shown by Nunes et al. [27] and Fernandes et al. [28]. *α* and *β* subunits of heterotrimeric G proteins are induced during the mycelium-to-yeast switch, and farnesyltransferase inhibitors (which disrupt Ras protein function by avoiding its correct membrane association) promote yeast-to-mycelium transition, respectively. Thermal dimorphism in *P. brasiliensis* is also found to be closely associated with the calcium signaling pathway through the heat shock protein 90 (HSP90), which binds and stabilizes calcineurin, thus controlling the cell differentiation [29]. *Pbhsp90* is a single-copy gene that reaches a 25-fold relative induction at one hour after mycelium-to-yeast transition, indicative of its participation upon a thermo-dependent response. Its expression was also found to be strongly induced under oxidative stress. Treatment with geldanamycin and radicicol, specific HSP90 inhibitors that affect the protein's ATPase activity, was shown lethal to the yeast cell in a dose-responsive manner, enforcing the potential of HSP90 as a target for novel antifungal therapies [30]. Further work using antisense technology demonstrated that PbHsp90 function is essential to *Paracoccidioides* physiology [31]. PbHSP90 plays a relevant role not only upon oxidative injury but also during growth in acid environment, which correlated with yeast cell viability 3 h postinteraction with activated macrophages, indicating that this protein increases the fungus' capability to adapt to the host.

On the other hand, several studies have shown that estrogens, specifically 17*β*-estradiol (E_2_), impair *P. brasiliensis* morphological transformation of the mycelial to the yeast form, which may explain the strong gender differences among adult population [32–35]. The exact mechanism involved in such modulation remains unclear; however, further analysis of this phenomenon using microarray technology revealed a correlation between estradiol, cell wall remodeling, energy metabolism, and cell signaling during the mycelium-to-yeast transition [36]. This study showed that as a response to overcome the presence of E_2_, the fungus delays or alters normal cellular responses triggered by high temperature, thus affecting subsequent morphological changes that compromised fungal adaptation and pathogenesis.

Simultaneously to the thermotolerance dimorphism, pioneer studies analyzing expressed sequence tags (ESTs) of cDNA libraries from *Paracoccidioides* spp. allowed to identify differently expressed genes during the mycelium-to-yeast transition and various host-interaction conditions, thus revealing the genus *Paracoccidioides*' specific metabolic adaptations intimately related to its environment [27, 37–40]. Moreover, initial proteomic approaches performed in the members of the *Paracoccidioides* spp. [41–43] characterized proteins expressed at their morphological phases and upon interaction with macrophages, reinforcing the complex multifaceted response mount by these fungi to facilitate their survival within the host and even modulate macrophages. As might be expected during the dimorphic transition [41], these authors showed preferential expression of proteins involved in the metabolism of amino acids, nitrogen, signal transduction, and several heat shock/stress-related proteins, including HSP88, HSP90, and isoforms of HSP70, consistent with the previous transcriptional analysis [27]. Notably, the enzymes transaldolase and transketolase are induced during the mycelium-to-yeast transition, indicating an upregulation of the pentose phosphate pathway, linked to the production of intermediates (fructose 6P and glyceraldehyde 3P) and recycling of NADP+ to NADPH, which are subsequently used by the yeast cell to produce ATP under anaerobic conditions. Altogether, these transcriptional and proteomic analyses set a starting point for integrative approaches on infection mimicking conditions to gain better knowledge about the interplay between the expression of microbial components, focusing on adapting/tolerating a harsh environment, and the host immune system (mainly macrophages), which produces profuse ROS and RNS activating antimicrobial activities to kill the fungal pathogen.

It is known that in the lungs, inhaled microorganisms are quickly phagocytized by macrophages supported by neutrophils and dendritic cells. Particularly, macrophages are considered a glucose- and amino acid-depleted environment; thereby, *Paracoccidioides* spp. have evolved defense mechanisms to survive under nutrient deprivation. Lima et al. [44] determined *P. lutzii* response in the absence of glucose performing a high-resolution transcriptomics and proteomic approach on cultured yeast cells and recovered yeast cells after macrophage internalization. The transcriptome analysis showed that under carbon starvation stress (6 h of carbon starvation), abundance of specific transporters such as those for copper, hexoses, and monosaccharides was augmented, indicating that carbohydrate, amino acid, and metal uptake processes are required for survival. Additionally, the ability to respond to oxidative stress was also demonstrated under carbon deprivation, since cellular responses against ROS such as superoxide dismutase, catalase, and cytochrome c peroxidase were elevated. In agreement with the transcriptome analysis, the proteomic response to carbon starvation involved an increase of proteins associated with metabolism (amino acid degradation, ß-oxidation, and ethanol production) and reduction of those related to core cellular processes (fatty acid biosynthesis). This study demonstrated how carbon-starved yeast cells modulate their metabolism by induction or repression of cellular activities. Overall data presented by these authors reveals that *P. lutzii* undergoes a global metabolic switch towards gluconeogenesis and ethanol production supported by precursors (acetyl-CoA, pyruvate, oxaloacetate, and succinate) from ß-oxidation, tricarboxylic acid (TCA), and glyoxylate cycles as a mechanism to adapt to carbon-starving conditions and survive in the hostile environment during macrophage infection.

Also, *P. brasiliensis* overcome the cell-mediated immune system by regulating morphogenesis. This can be achieved by a fungal Rho GTPase, Cdc42, which is involve in controlling actin-mediated polarized growth and supports the large size of the yeast cell and its multibudding state, a morphology that inhibits phagocytosis [45]. Indeed, RNAi *cdc42* strains are more efficiently phagocytosed by macrophages and display decreased pathogenicity [45].

### 2.2. Changes in Cell Wall Polysaccharide Composition

**Figure 1:**
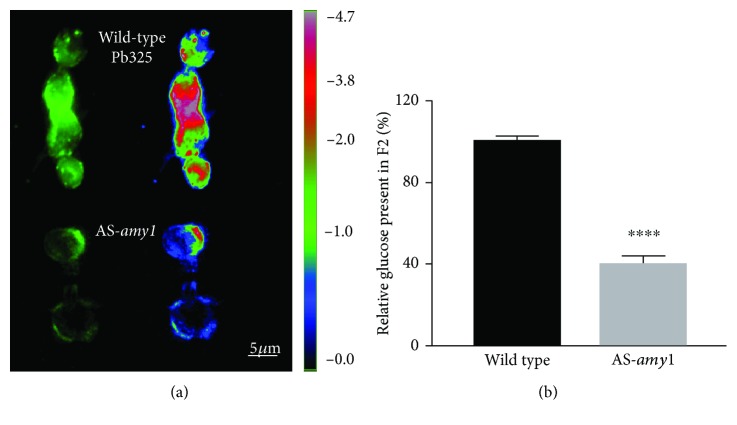
Silencing of Pb*AMY1* reduces *α*-(1,3)-glucan of *P. brasiliensis* yeast cell. (a) Semiqualitative estimation of *α*-(1,3)-glucan on *Paracoccidioides* yeast cells by immunofluorescence. Pseudocolor mask for saturation (ImageJ). (b) Quantification of *α*-(1,3)-glucan in *Paracoccidioides* yeast cells by anthrone assay. ^∗∗∗∗^*P* < 0.00001, Welch's test.

*Paracoccidioides* spp. are characterized by a distinctive structure and chemical differentiation in its cell wall components per the morphological phase in which it stands at a given moment. While the mycelial phase cell wall has *β*-1,3-glucan as the main neutral glucose polymer, the multibudding yeast-like phase reduces this polysaccharide to a minimum and substitutes it by *α*-1,3-glucan (Table 1; [46]), a change that has been correlated with pathogenicity, since spontaneous loss of the polysaccharide correlated with decreased virulence [47]. This initial observation relating *α*-(1,3)-glucan as a fungal virulence factor was demonstrated 30 years later in *H. capsulatum* [48]. The presence of *α*-(1,3)-glucan in the outermost layer of the cell wall of *H. capsulatum* yeast masks *β*-(1,3)-glucan, an immunogenic component of fungal cell walls, avoiding its recognition from pattern recognition receptors (PRR) found on host phagocytic cells [48]. Disturbance of the *α*-(1,3)-glucan synthesis by depletion of the *H. capsulatum α*-(1,4)-amylase (*AMY1*) transcript, which is involved in priming the oligosaccharide synthesis, reduces cell wall *α*-(1,3)-glucan content and fungal virulence [49, 50]. Preliminary data from immunofluorescence and biochemical studies after silencing *P. brasiliensis AMY1* (Pb*AMY1*) showed 60% reduction of the *α*-(1,3)-glucan content on AS-*amy1* yeast cell wall, indicating that indeed PbAMY1p plays a relevant role in the *Paracoccidioides* spp. *α*-(1,3)-glucan synthesis (Figure 1). In yeast cells, the drastic reduction of the immunogenic polysaccharide *β*-(1,3)-glucan in its cell wall, and its substitution by *α*-(1,3)-glucan as an outermost layer when compared to the mycelial phase [51], might be an evolutive feature, hampering the recognition of the yeast cell by the phagocytic cells of the host, as in *H. capsulatum* and, therefore, acting as a protective shield against host defense.

It has been reported for *P. brasiliensis* that the relative content of cell wall polysaccharide is not a constant when different strains are compared and could vary not only with culture conditions but also among isolates [52–54]. However, common features still stand: in the mycelial phase, *β*-1,3-glucan is present as the mayor structural polysaccharide in different strains, regardless of the phylogenetic group to which each strain belongs, while in the yeast form, *α*-(1,3)-glucan is present as the mayor neutral polysaccharide and chitin as the mayor structural polysaccharide (Table 1). These features also apply to a single strain growing on different culture media (Table 2).

Furthermore, it is also well known that long periods of successive subculturing of *Paracoccidioides* spp. lead to attenuation or loss of virulence due to compositional changes of the cell wall [55, 56], which can be re-establish after passage in animals [47], or epithelial culture cells [57] or by supplementing culture media with growth factors such as fetal calf serum [52]. Nevertheless, a biochemical study of *P. brasiliensis* and *P. lutzii* cell wall composition in the presence of horse serum showed interesting differences among them (Table 3). *P. lutzii* showed no increase in its *α*-(1,3)-glucan content after growth in the presence of horse serum while *P. brasiliensis* did, reinforcing the role of molecular evolution in microbial attributes associated with virulence of these two organisms.

**Figure 2:**
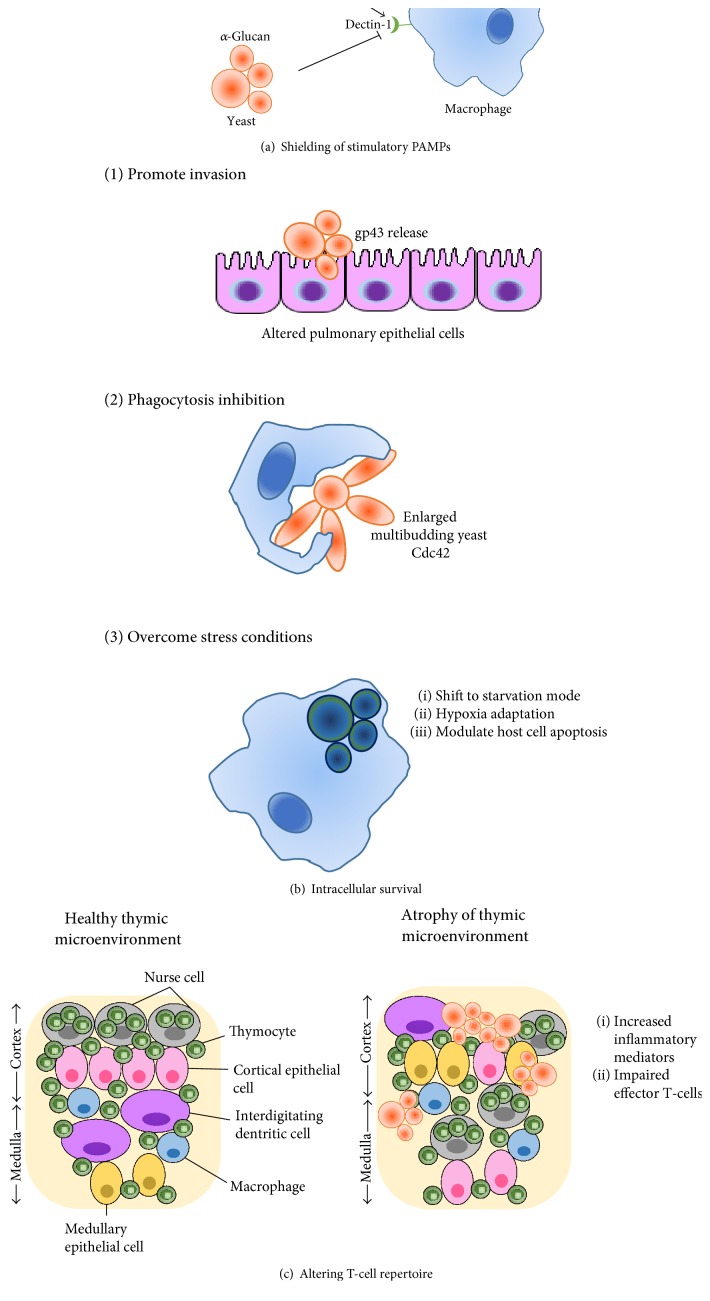
Diagram of proposed *Paracoccidioides* spp. immune-evasion mechanisms. (a) Shielding of stimulatory PAMPs. The cell wall beta-glucan present in the fungus saprophytic forms (conidia and mycelia) is recognized by the macrophage Dectin-1 receptor; however, pathogenic yeast cell *α*-(1,3)-glucan masks *β*-(1,3)-glucan, avoiding its recognition. (b) Intracellular survival. *Paracoccidiodes* spp. use several strategies to overcome the host harsh environment, among them are the following: (1) promoting invasion to the pulmonary epithelial cells by altering their cytoskeleton structure, a process assisted by gp43; (2) avoiding phagocytosis by displaying an enlarged multibudding morphology, boosted by Cdc42 expression, which physically impairs engulfment by macrophages; and (3) adapting to the host environment. The phagocytosed fungus shifts its metabolism to tolerate macrophage stress conditions and even modulate host apoptosis enabling fungal killing. (c) Altering T-cell repertoire. During acute fungal infections, yeast cells invade the thymus altering its epithelial cells' spatial arrangement crucial for T-cell differentiation and pathogen-specific immune response.

Cell wall turnover during infection after morphological switching is a survival strategy used by dimorphic fungi to avoid recognition by the PRRs of the host phagocytic cells. Dectin-1, a PRR present on the surface of host phagocytic cells, recognizes fungal cell wall *β*-(1,3)-glucan and triggers phagocytosis, respiratory burst, and release of cytokines such as TNF-*α*, IL-12, and other interleukins. The spatial arrangement of the yeast cell wall *α*-(1,3)-glucan in *Paracoccidioides* spp. and *H*. *capsulatum*, present as an outermost layer, covering the immune stimulatory PAMP *β*-(1,3)-glucan, could actively interfere with these events (Figure 1(a)). A molecular study made for *H. capsulatum* revealed that silencing macrophage Dectin-1 gene expression suppressed the production of proinflammatory TNF-*α* by phagocytes, suggesting that *α*-(1,3)-glucan effectively shields *β*-(1,3)-glucan from innate immune recognition by the Dectin-1 receptor [48, 52] (Figure 2(a)). Additionally, a recent study in *α*-glucan-containing *Histoplasma* strains showed that yeast cells of this organism secrete an endo-*β*-(1,3)-glucanase, Eng1, which plays a role in fine scale hydrolysis of cell wall *β*-glucans [58]. Eng1 acts trimming *β*-glucan segments exposed on the fungal cell surface further minimizing potential Dectin-1 recognition, decreasing production of proinflammatory cytokines by phagocytes thereby enhancing *Histoplasma* ability to escape detection by host phagocytes. Interestingly, two endoglucanases associated with *P. brasiliensis* extracellular proteome have been reported [59]; however, none shows homology to *Histoplasma* Eng1.

## 3. Adhesion and Invasion

As the infection process advances through the respiratory pathway, *Paracoccidioides* spp. are required to cross tissue planes aiming their intracellular persistence within the host; therefore, the fungus initially invades normally nonphagocytic host cells such as epithelial cells and endothelial cells inducing their own uptake and causing host cell apoptosis [60–62].

### 3.1. Adhesins

At this stage, *Paracoccidioides* spp. surface proteins known as adhesins play a critical role in the establishing of the infection by interacting with the host cells to promote successful colonization and/or dissemination of the fungi into the host organism [63]. Adhesins mediate fungal cell binding to host extracellular matrix (ECM) components (mainly fibronectin, laminin, fibrinogen, type I and IV collagen, and plasminogen) as well as to epithelial lung cells [64]. Differences in adhesion capacity to Vero cells [65], pneumocytes, and ECM components [66] have been observed for *Paracoccidioides* spp., which might also be attributable to changes in the cell wall composition [67]. Several studies have allowed to identify a diverse number of adhesins in *Paracoccidioides* spp., which are involved in the interaction with host cells and in the in vitro biofilm formation, revealing this fungus a high level of adaptability to a new environment (reviewed by [68]) [63, 69, 70].

A surface glycoprotein of 43 kDa, the first adhesin described in *P. brasiliensis* known as gp43, showed adhesion to laminin and fibronectin [71, 72]. It was the first adhesin to be reported as enhancer of pathogenesis in this fungus. Gp43 inhibits both phagocytosis and fungal intracellular killing [73], may induce protection depending on the route of infection [74], and strongly induces in vitro granuloma-like formation by B-1 cells and macrophages [75]. Downregulation of Pb*GP43* correlated with reduced fungal burden in the lungs of the infected BALB/c mice [76]. Gp43 is likely to be found within vesicles [59] and also happens to be the predominant antigen used for immune detection of *P. brasiliensis* [2]. In the case of *P. lutzii*, a gp43 ortholog, named Plp43, shares only few epitopes in common; therefore, gp43 should not be used in the diagnosis of PCM patients infected with *P. lutzii* [77]. PbHad32p, a 32 kDa protein member of the hydrolase family, able to bind to laminin, fibronectin, and fibrinogen, has been shown to be important in the initial attachment of the infectious particles to the lungs [78–81]. Once into the host, *Paracoccidioides* spp. infective propagules switch to yeast cells, which manage to ease their invasion into pulmonary epithelial cells and keratinocytes by altering the host cell cytoskeleton structure, a process that is promoted by gp43, which acts as an adherence receptor in the internalization of the yeast into the host cell (Figure 2(b)). When into the pulmonary epithelial cell, the fungus induces cytokeratin degradation and apoptosis of the host cells [61, 67].

A phospholipase B (PLB), involved in the early fungus-macrophage interaction, has been reported crucial during the invasion of the host by *Paracoccidioides* spp. and suggested to possibly modulate the innate immune response [82]. Many other potential adhesins, previously described as upregulated genes in yeast cells derived from models of infection, have been uncovered by a comparative transcriptome analysis of annotated ESTs during in vitro adherence assays to type I collagen and fibronectin, including C-5 sterol desaturase, cap20 protein, high-affinity copper transporter, hexokinase, and transketolase [37, 39, 83].

Another set of surface adhesins well characterized as moonlighting proteins in *Paracoccidioides* spp. includes enolase (ENO), fructose 1,6 bisphosphate aldolase (FBA), glyceraldehyde 3-phosphate dehydrogenase (GAPDH), triosephosphate isomerase (TPI), malate synthase (MLS), isocitrate lyase (ICL), and aconitase (ACO) (reviewed by [69]). These are multifunctional proteins that can perform several additional functions, besides their role in chemical metabolic reactions. Most likely, moonlighting proteins act as enzymes constitutively expressed at low levels, but when performing moonlighting functions, they are expressed at high levels [84].

*Paracoccidioides* spp. ENO, FBA, GAPDH, and TPI are glycolysis enzymes that have been detected in the fungus surface as well as in the vesicle proteome [59], in addition to their conventional cytoplasmic localization. ENO is a 54 kDa protein that binds to laminin, fibronectin, plasminogen, and type I and IV collagen [85, 86]. PbEno expression reported a 10-fold increment on yeast cells derived from the lungs, livers, and spleen of mice after 7 days. Heterologous expression of *Paracoccidioides* enolase (r*Pb*Eno) allowed to evaluate its role in the infection of host cells, suggesting that r*Pb*Eno promoted an increase in the association (adhesion/invasion) of *Paracoccidioides* spp. with host cells in ex vivo models of infection [87]. PbEno's ability to bind plasminogen seems to favor the yeast cell attachment and internalization to host tissues by modifying the surface of host cells (degradation of fibronectin), therefore playing a key role in the establishment of PCM [83, 87]. The enolase plasminogen-binding ability and its role in the degradation of host tissues and ECM components also have been related to the invasion process in *Plasmodium* parasites and other pathogens [88]. A proteomic analysis of *P. lutzii* secretome allowed the identification of fifteen plasminogen-binding proteins, among them is FBA [89]. FBA's ability to bind plasminogen increased fibrinolytic capacity of the fungus, as demonstrated in the fibrin degradation assay. Its participation in the host-pathogen interaction was also evaluated using recombinant protein or anti-FBA antibody in which reduction of adherence/internalization by macrophages was demonstrated [89]. The GADPH binds to laminin, fibronectin, and type I collagen. Its expression is increased during the mycelium-to-yeast transition and parasitic yeast phase; thus, it seems to be involved at the early stages of the fungal infection promoting adhesion to host tissues. In vitro assays treating *Paracoccidioides* spp. yeast cells with polyclonal anti-GAPDH antibody or pneumocytes with the recombinant protein demonstrated reduced interaction between the host and fungus [90, 91]. TPI was initially described as a fungal antigen able to react with the sera of PCM patients [92]. Further characterization and production of an antirecombinant TPI (r*Pb*TPI) polyclonal antibody showed TPI role as an adhesin, which binds preferentially to laminin, and it is involved in the initial fungal adherence and invasion [93].

MLS and ICL are key enzymes of the glyoxylate cycle required for fungal virulence [94]. In *Paracoccidioides* spp., their transcript levels are induced during the mycelium-to-yeast transition and the yeast cell [25, 95], particularly during nutritional stress conditions. MLS is upregulated in yeasts during phagocytosis by macrophages [96], while ICL during the fungus-macrophage interaction upon carbon starvation [44], suggesting their relevance for infection. MLS also participates in the allantoin degradation pathway, which allows the cells to use purine as a nitrogen source [97]. PbMLS also showed differential accumulation and reactivity on *Paracoccidioides* spp. surface and cytoplasm of budding cells, respectively, and not in the mother cell, indicating that this enzyme is metabolically relevant and mainly synthesized by young cells. The recombinant protein demonstrated ability to recognize fibronectin and type I and IV collagen, as well as pulmonary epithelial cells, implying PbMLS involvement in the interaction of the fungus with host components [98]. ICL binds fibronectin, type IV collagen, and epithelial cells; it is also secreted to the fungal surface [99], supporting the protein relevance during the host-pathogen interaction. Notably, *Pb*ICL is regulated by carbon sources, and its inhibition by argentilactone, a natural drug previously used in the experimental treatment of cutaneous leishmaniasis, can affect cell growth and differentiation [100].

ACO is involved in energy generation catalyzing the isomerization of citrate to isocitrate in both the TCA cycle and the glyoxylate cycle. *Paracoccidioides* spp. ACO (*Pb*ACO) is a 80 kDa protein found in the extracellular fluid, preferentially expressed in yeast cells associated with cell wall, mitochondria, cytosol, and peroxisomes. *Pb*ACO protein levels in yeast cells were induced when fungal growth used potassium acetate or ethanol as carbon sources and in the presence of high-iron concentrations, indicating a potential role in iron metabolism [101].

Furthermore, a 30 kDa adhesin also identified as a 14-3-3 glycoprotein might also be considered a moonlighting protein in *Paracoccidioides* spp. [102]. Initial studies of *P. brasiliensis* 14-3-3 protein showed that it preferentially binds to laminin and presented evidence that adhesion capacity could be related to virulence [57]. 14-3-3 is localized in both the cytoplasm and the cell wall [59]; however, its concentration on the cell wall largely increased during infection, stressing that 14-3-3 plays an essential role in the host-pathogen interaction [103]. Functional analysis of *Pb14-3-3* in *Saccharomyces cerevisiae* partially complemented Bmh1p and Bmh2p proteins supporting the role as an adhesin and demonstrating reduced susceptibility to fluconazole in *S. cerevisiae* transformants [104]. This study shows that Pb14-3-3 might be involved in the ergosterol biosynthesis revealing a potential new drug target. Recent work silencing *Pb14-3-3* distinctly altered the yeast morphology and hampered the morphological switching without affecting cell vitality or viability [105]. Additionally, these authors demonstrated that binding of the *Pb14-3-3* mutant to laminin and fibrinogen was reduced compared to that of the control, which correlated with a significant reduction of the virulence phenotype in the invertebrate infection model *Galleria mellonella.* This study established multifaceted roles of *Pb14-3-3* in morphology, attachment/infection to host components, and virulence, therefore supporting the previous report that suggested 14-3-3 as interesting therapeutic target for the treatment of PCM. Further intracellular survival and dissemination of *Paracoccidioides* spp. is accomplished by modulating programmed cell death of macrophages and epithelial cells, through expression of caspase-2, caspase-3, and caspase-8, strongly influenced by the 30 kDa 14-3-3 and gp43 adhesins [106, 107].

## 4. Defenses to Host Environment Stressors

Upon successful invasion of the mammalian host and reaching an intracellular niche, *Paracoccidioides* spp. require to overcome environmental stressors, persist intracellularly, and manipulate the progression of disease in the host. Other microbial determinants of *Paracoccidioides* spp. that play a relevant role in their pathogenesis are the following.

### 4.1. Melanin

Melanin pigments are remarkable substances present in all biological kingdoms, which have been associated with myriad functions based upon their unique physicochemical properties (reviewed by [108]) [109]. Melanins are polymers of phenolic and/or indolic compounds, negatively charged, hydrophobic in nature, and with high molecular weight and unknown structure [110]. Most fungi, bacteria, and helminths synthesize melanin via the polyketide synthase pathway or catalyze it by phenoloxidases (reviewed by [111]). Particularly in the field of fungal pathogenesis, their role in virulence has been well established (reviewed by [112–115]). In *Paracoccidioides* spp., melanin characterization was first described by Gomez et al. [116]. These authors revealed *P. brasiliensis*' ability to produce melanin when recovering dark particles that retained the size and shape of conidia or yeast, after enzymatic digestion and harsh acid treatment. Interestingly, melanized conidia were obtained after growing mycelia on water agar, while melanized yeasts were observed during growth in minimal media supplemented with L-3,4-dihydroxyphenylalanine (L-DOPA) and also recovered from infected mouse tissue, indicating the fungus capacity to synthesize melanin in the absence or presence of L-DOPA. Data also demonstrated that melanized yeast cells, either grown in vitro or recovered from infected tissue, were reactive to melanin-binding monoclonal antibodies (MAbs) isolated from *Cryptococcus neoformans* [117], showing consistency with in vivo melanization. Analysis by electron spin resonance (ESR) spectroscopy of *Paracoccidioides* spp. melanin recovered from yeast cells demonstrated a strong signal characteristic of a stable free-radical population, a key criterion in defining a melanin. Moreover, melanin synthesis by yeast cells was supported by the presence of laccase activity in cytoplasmic extracts. Additionally, upregulation of genes related to melanin synthesis such as tyrosinase and aromatic L-amino acid decarboxylase was shown in infected mice [37].

Fungal melanin distribution varies among species, for example, *Candida albicans* melanin can be found in the outer part of the cell wall and/or clustered on the cell wall surface [118], while in *C. neoformans*, melanin is first detectable close to the plasmatic membrane and fills throughout the cell wall over time [113]. Using transmission electron microscopy, *Paracoccidioides* spp. melanin was shown as electron-dense granules distributed on the yeast cell surface as well as in the cytoplasm [119]. Latest studies about cryptococcal melanin revealed that this polymer is composed of granular particles with an average size of 75 nm in diameter [120, 121]. Moreover, melanin synthesis takes place within laccase-containing vesicles known as fungal melanosomes [122], which might interact with cell wall components such as chitin to facilitate melanin deposition within the cell wall [123–126].

MAbs against *Paracoccidioides* melanin have been generated [127]. This study reported that the melanin-binding MAbs (IgG and IgM) successfully labeled conidia from mycelial cultures grown in water and yeasts grown in the presence of L-DOPA, as well as condium-infected mouse lung tissue. Melanin production during PCM was demonstrated by the detection of IgG Abs in serum specimens from patients; however, sera from patients with different mycoses displayed cross-reactivities against a wide spectrum of fungal melanin types, which supports the hypothesis that melanin may represent a “common” or immunological target for pathogenic fungi [128]. Antibodies to fungal melanin have provided protection against *C. neoformans* [129] and *Fonsecaea pedrosoi* [130].

Initial studies concerning melanin capacity to protect *P. brasiliensis* yeast cells from the host immune system reported that mannan can partially inhibit phagocytosis and that melanized cells were more resistant than nonmelanized cells to fungicidal and fungistatic effects of macrophages; however, increased macrophage uptake of opsonized yeast cell was documented when adding complement and/or antibody against melanin [119]. Further analyses on this area investigated the effect of *P. brasiliensis* melanized yeast cells on antimicrobial oxidants and phagocytosis using carbohydrates and monoclonal antibody to CD18 [131]. This study showed significant reduction in the phagocytosis of melanized yeast cells by macrophages, previously treated with mannan or laminarin; moreover, phagocytosis was virtually abolished when phagocytic cells were treated with mannan and *N*-acetylglucosamine in the presence of anti-CD18 antibodies, suggesting that macrophage internalization of melanized yeasts requires multiple receptors. In vitro analyses demonstrated that melanized cells were less susceptible to chemically generated nitric oxide, oxygen-derived oxidants, chloride-free sodium hypochlorite, and to killing by hydrogen peroxide than nonmelanized cells. These data correlated with an infection in a murine model, which resulted in increased fungal burden in the lungs by melanized yeast compared to nonmelanized cells, most likely attributable to reduced internalization by phagocytic cells and enhanced resistance to intracellular death. Therefore, melanin promotes fungal virulence by inhibiting phagocytosis and neutralizing oxidative radicals generated in the host effector cells.

Furthermore, in search for an alternative treatment to PCM skin lesions and oral mucosa, the influence of melanin produced by sixteen isolates of the *Paracoccidioides* spp. complex on the effects of treatment with antimicrobial photodynamic inhibition (aPI) and antifungal drugs was evaluated [132]. These authors demonstrated that aPI can reduce the viability of *Paracoccidioides* spp.; however, melanized yeast cells were more resistant than nonmelanized cells, which was attributable to lower levels of ROS and RNS due to melanin interference with the absorbance peak of toluidine blue. In addition, MIC data showed that melanized yeast cells were less susceptible to amphotericin and itraconazole, while in the previous study, da Silva et al. [119] found no differences between melanized and nonmelanized yeast cells. Nevertheless, studies from da Silva et al., using an antifungal killing assay for melanized yeast cells of *Paracoccidioides* spp., revealed increased resistance to antifungal drugs mainly amphotericin B and less pronounced with ketoconazole, fluconazole, itraconazole, and sulfamethoxazole, which could be thought to be attributable to reduced cell wall permeability or that melanin quenched free radicals released by cell membrane damaged by drugs [133].

Interestingly, studies by Baltazar et al. [132] showed that melanin can interact with amphotericin, itraconazole, and toluidine blue, consequently changing their antifungal activities. Other authors have demonstrated that melanin binds amphotericin and not itraconazole by analyzing the elemental composition of C:N:O after incubation of these drugs with melanin [134], suggesting that melanin alters the drug composition; however, Baltazar et al. reported that melanin might physically block itraconazole entrance to the yeast thus reducing its activity, while decrease in the antifungal activity of amphotericin is due to the alteration of the drug structure that reduces its affinity for ergosterol. Altogether, these data confirm that melanization contributes to virulence by acting as a ROS scavenger and through binding to antifungal drugs, thereby altering their activities [119, 131, 132].

### 4.2. Extracellular Vesicles

Fungal extracellular vesicles (EVs) resembling mammalian exosomes have been reported (reviewed by [135–139]). So far it is known that EVs, using a noncannonical pathway of secretion, are able to cross the cell wall and transport molecules that play a role in nutrient acquisition, cell defense, and even modulation of the host immune defense; however, many questions about their biogenesis, mechanisms through which EV transverse the cell wall and reach the extracellular space, and how they modulate host interactions remain to be elucidated. Nevertheless, the compositional analysis of such EVs present in the fungal pathogens *C. neoformans*, *H. capsulatum*, *C. albicans*, *Candida parapsilosis*, and *Sporothrix schenckii* suggests that they might act as “virulence bags” [140]. In fact, it is reported that in *C. neoformans*, glucoronoxylomannan (GXM), the major capsular polysaccharide, is transported within vesicles to the extracellular space where it is released and reincorporated into the cell surface as an alternative pathway for capsule growth [141]. These extracellular compartments composed of lipid bilayers have the potential to regulate key pathogenic steps during fungal infections. Particularly in the genus *Paracoccidioides*, a pioneer study characterized EVs isolated from culture supernatants of *P. brasiliensis* yeast cells cultivated in defined media [42]. This study demonstrated that the fungus EVs carry antigenic components bearing highly immunogenic *α*-galactopyranosyl (*α*-Gal) epitopes, which were found both at the vesicle surface and at the lumen. Both PCM and chagasic anti-*α*-Gal IgG reacted intensely with EVs, in contrast with the slight reaction evoked by natural anti-*α*-Gal antibodies, thereby suggesting that in *Paracoccidioides* spp., there is a high variety of nonreducing terminal *α*-linked galactopyranosyl epitopes that may resemble those found in *Trypanosoma cruzi* mucins.

Furthermore, a unique proteomic analysis of EVs and vesicle-free released proteins from *Paracoccidioides* spp. pathogenic yeast phase provided a comparative analysis with other pathogenic fungi EV proteomes [59]. This study identified 205 and 260 proteins in vesicle and vesicle-free preparations, respectively. According to their sequences, almost 70% of them were predicted secretory, mostly involved in nonclasical secretory pathways. The comparative analysis of *Paracoccidioides* EV proteins with orthologs present in vesicles from *C. neoformans*, *H. capsulatum*, and *Saccharomyces cerevisiae* revealed that 63% of the *Paracoccidioides* vesicle-associated sequences had orthologs in other fungal extracellular vesicles, and among them, 72 were common to *Paracoccidioides* spp. in at least two other species, while 26 were identified in all four species analyzed. Some of these proteins might have clear roles during infection, for instance, superoxide dismutase, mitochondrial peroxiredoxin, and thioredoxin, which are involved in the ROS homeostasis and promote fungal intracellular survival. Interestingly, this analysis also revealed that the composition of the secretome is strongly affected by the growth conditions, suggesting that adaptation and survival to certain environments are closely associated with the profile of released proteins. Overall, it was reinforced with this study that EV cargo is complex, and it might involve proteins with diverse physiological functions from signaling to cell division to response to stress.

Concerning the complexity of fungal EVs, da Silva et al. [142] demonstrated that mannose and *N*-acetylglucosamine residues are found in *Paracoccidioides* EV surface, which are recognized by the innate immune system receptors DC-SIGN and DC-SIGNR, but not Dectin-1 or Dectin-2. Moreover, the influence of EVs produced by *P. brasiliensis* yeast cells on the host immune cells was evaluated [143]. These authors showed that incubation during 48 h of EVs and murine peritoneal macrophages induced the release of proinflammatory mediators such as NO, IL-12p40, IL-12p70, IL-6, TNF-*α*, IL-1*α*, and IL-1ß in a dose-dependent manner. Similarly, it was shown that EVs promote a proinflammatory profile in murine macrophage J774A.1 cells. Additionally, it was demonstrated with this study that EVs favor the development of macrophages towards the “classical” M1 activation phenotype, and even more, *Paracoccidioides* EVs can stimulate macrophage switching from an M2 towards an M1 phenotype. Remarkably, EV-stimulated macrophages, during 24 h, exhibited a higher fungicidal activity than those macrophages activated with IFN-*γ*, which was evident by the lower recovery of yeast CFU from lysed macrophages. Therefore, this study suggests that EV component from *Paracoccidioides* spp. can modulate the host immune response and affect the interplay of fungus-host immune cells.

#### 4.2.1. How Paracoccidioides Spp. Overcome Host Environmental Stressors?

Macrophage oxidative burst is characterized by increased oxygen uptake and ROS production that along the release of hydrolytic enzymes and toxic metabolites inside the phagolysosome intend to kill fungal pathogens. Nitrosative molecules (such as nitric oxide), produced mainly by INF-*γ*-activated macrophages, are fungicidal to *Paracoccidioides* spp. [144, 145]. *Paracoccidioides* complex initiates a metabolic switch to tolerate the macrophages' carbon-depleted environment, particularly by activating the pentose phosphate pathway, which additionally provides a defensive mechanism to the yeast cells against sulfhydryl groups and oxygen radicals from the host by maintaining glutathione in a reduced state [146]. Moreover, high-throughput transcriptional and proteomic analysis studies in *Paracoccidioides* spp. revealed that upon macrophage phagocytosis [147, 148], mimicking oxidative stress by exposure of yeast cells to H_2_O_2_ [149], or inducing nitrosative stress to yeast cells by incubation with S-nitrosoglutathione (GSNO) [150], which produces RNS, the fungus can cope with oxidative and nitrosative stress. In response to H_2_O_2_, *Paracoccidioides* spp. present a prominent activation of antioxidant enzymes (catalases, cytochrome c peroxidase, thioredoxin, and superoxide dismutases) and induce a metabolic shift to the pentose phosphate pathway, characterized by increased NADPH production in the cytoplasm as an electron source for glutathione peroxidase system, in order to restore the cellular redox potential [149]. This data correlates with the upregulation of transcripts of genes encoding peroxisomal catalase and Mn superoxide dismutase in yeast cells infecting macrophages associated with glucose and amino acid limitation [147]. Other studies evaluated the role of an alternative respiratory chain (AOX) in *Paracoccidioides* spp. during host-pathogen interaction [151, 152], which has been shown to be involved in the control of ROS and other oxidative molecules [153, 154]. Through generation of a knockdown strain PbAOX-aRNA, these authors demonstrated reduced fungal viability during infection of alveolar macrophages, particularly during the morphological transition, thus decreased fungal burden in the lungs of infected mice and increased survival rate. These data support that PbAOX is essential during the establishment of the fungal infection, possibly by assisting redox balancing during cell growth and the morphological switch of *Paracoccidioides* spp.

Understanding *Paracoccidioides* yeast cell behavior to nitrosative stress was achieved by identifying genes and proteins that might contribute to this response; this study demonstrated reduced levels of enzymes related to aerobic respiration (specifically cytochromes, succinate dehydrogenase, and ATP synthases), indicative of reduced activity of the mitochondrial electron transport chain [150]. In the presence of GSNO, these authors also reported alterations in lipids and branched chain amino acid metabolism and noticed increased expression of the enzymes cytochrome C peroxidase (CCP) and superoxide dismutase (SOD), which have been involved in *Paracoccidioides* spp. oxidative stress response [149]. Consequently, the overlapping role of CCP and SOD in both stress responses was confirmed by knockdown approaches [148, 150, 155]. *Paracoccidioides* spp. *ccp*-aRNA strains are more sensitive to RNS [150] and mitochondrial-generated ROS stress [148], suggesting that CCP avoids cell damage caused by nitrosative and oxidative stress. Additionally, these authors reported that CCP silencing promoted a reduction in the number of recovered fungi in macrophages and in an animal model, thereby CCP can be considered a virulence factor since it is relevant for the establishment of the infection by *Paracoccidioides* spp. [148]. Another study has reported *Paracoccidioides* spp. ability to reduce nitric oxide (NO) levels by secreting the adhesin gp43, which prevents the release of NO from macrophages and stimulates the release of IL-10, hence reducing the iNOS expression and its enzymatic activity [73].

Concerning SOD role in the response to oxidative stress, Tamayo et al. [155] identified and characterized six isoforms encoded in the *P. brasiliensis* genome, among which Pb*SOD1* and Pb*SOD3* expressions were increased during the morphological switching to the pathogenic yeast phase, as well as under treatment with oxidative agents and during interaction with phagocytic cells (PMNs and alveolar macrophages). Interestingly, as shown by these authors, silencing of Pb*SOD1* and Pb*SOD3* genes has no detrimental effect on yeast cells' growth rate; however, both knockdown strains were similarly susceptible to H_2_O_2_- and menadione-induced oxidative stress, while PbSod3p was required for virulence. This study propose a well-coordinated response to oxidative stress in *Paracoccidioides* spp., in which intracellular Sods (mostly Sod1p) defense against endogenous-produced ROS while Sod3p, supported by its extracellular activity and cell surface localization, assists in combating the superoxide radicals generated during the host-pathogen interaction.

Furthermore, Tamayo et al. [156] recently identified and characterized the three members of the catalase (CAT) gene family in different fungal strains of *Paracoccidioides* spp., covering each phylogenetic lineage, as well as in other Onygenales. This study revealed that *Coccidioides* and dermatophyte genomes do not encode the extracellular catalase CATB, suggesting that Onygenales may have evolved different mechanisms to counteract oxidative stress via catalases. Moreover, in correlation with the SOD study [155], yeast cells from *P. brasiliensis* showed higher expression of *CATP* than those of *P. lutzii*. Having a similar experimental strategy as in the SOD analysis, these authors demonstrated that PbCATA and PbCATB play a major role in endogenous ROS homeostasis in yeast cells, whereas PbCATP is mainly triggered in the presence of exogenous ROS and the reduced expression of this isoform negatively affected fungal virulence in a mouse model. The data shows that *Paracoccidioides* spp. rely on CAT isoforms to control ROS homeostasis along the different stages of the infectious process to promote fungal survival and virulence [155].

Iron and zinc are essential micronutrients in fungi, due to their participation as cofactors in many biological processes inside the cell. Therefore, host cells impede intracellular microbial proliferation by restraining access to iron through its sequestration by high-affinity iron-binding proteins, such as transferrin and ferritin. Particularly, under iron starvation conditions, a proteomic analysis of *Paracoccidioides* spp. yeast cells grown in media supplemented with the iron chelator bathophenanthrolinedisulfonate (BPS) showed potential repression of the TCA cycle which is mediated by enzymes containing Fe/S clusters, reduced expression of enzymes involved in the glyoxylate pathway and methylcitrate cycle, downregulation of the electron transport chain, and decreased in oxidative phosphorylation thus lowering ATP production. Overall, these data revealed that in response to iron deprivation, the fungus adjusts their energy metabolism to iron-independent pathways by increasing glycolytic activity thus compensating for the decrease of aerobic pathways [157]. Furthermore, in *Paracoccidioides* spp., it has been shown that host hemoglobin and siderophore production and transport are iron sources for the fungus [158, 159]. Hemoglobin uptake is mediated by the hemoglobin fungal receptor ortholog, Rbt5 [158]. A *rbt5* knockdown strain of *Paracoccidioides* spp. showed a lower survival rate inside macrophages and lower fungal burden in vivo in a mouse model of infection, a result which suggests Rbt5 as a virulence factor and a possible way to overcome low levels of iron by a highly effective iron uptake by the fungus [158]. Another way to accumulate intracellular iron, described in *Paracoccidioides* spp., is a nontraditional reductive iron assimilation (RIA) pathway, involving iron reduction and zinc-regulated transporter homologs (Zrt1 and Zrt2), able to transport zinc and iron inside the fungal cell [160, 161]. This would suggest that under stress conditions, like those found inside the macrophage, those *Paracoccidioides* spp. yeast cells successfully phagocyted by macrophages, could shift to a starvation mode, and activate highly effective iron and zinc uptake pathways, in order to persist inside the microenvironmental conditions of phagocytic cells.

It is established that in inflamed tissues, oxygen supply is limited by the high volume of host phagocytic cells or the microbe itself at blood vessels. *Paracoccidioides* spp. must tolerate and overcome stress conditions caused by low oxygen levels. Characterization of *P. lutzii* hypoxia response by a proteomic approach revealed differential protein expression for 134 and 154 proteins at 12 and 24 hours under hypoxy conditions when compared to the control [162]. At 12 hours under hypoxia, 50% of the proteins showing differential expression were increased, while the same percentage was decreased when compared to control cells, while at 24 hours under hypoxia, around 66% of proteins showing differential expression were increased, while around 33% were decreased. An evaluation of mitochondrial activity showed a lower activity at the first 12 hours under hypoxia, and restoration of activity at 24 hours, in agreement with the proteomic results, showing the potential for adaptation of this fungus under low oxygen levels [162]. The same work revealed that *P. lutzii* contains homologs of SrbA, a sterol regulatory element binding protein (SREBP) and key regulator of hypoxia adaptation in fungi [162]. Functional complementation of an *Aspergillus fumigatus srbA* null mutant by the *Paracoccidioides srbA* (*PbsrbA*) gene restored the null mutant hyphal growth under hypoxia, which suggests that *PbsrbA* may promote adaptation to hypoxic microenvironments. Furthermore, this study also showed that *Paracoccidioides* SrbA is likely involved in azole drug resistance responses. Perhaps this resistance could be achieved by regulating brassicasterol biosynthesis, which is found in *Paracoccidioides* spp. yeast cells' cytoplasmic membranes, instead of ergosterol [163], compensating the effects on membrane fluidity due to low oxygen levels.

## 5. Dissemination

On the last stage of the infectious process, the ability to establish the fungal infection in distant niches through biofilm formation represents a critical virulence factor in *Paracoccidioides* spp. A recent report showed that *P. brasiliensis* can colonize surfaces and form biofilms in its yeast phase [70]. The fungus biofilm consisted of a dense network of yeast cells characterized by the expression of genes encoding adhesins (gp43, GAPDH) and hydrolytic enzymes (aspartyl proteinase), consistent with the established steps of adhesion, invasion, and tissue destruction also reported for *C. albicans* biofilms [164]. Biofilm formation by the fungus might be a critical factor in the persistence of the fungal infection, since it could hinder the action of antifungal drugs and may contribute to a chronic state of the disease. Additionally, gp43 inhibits the phagocytic and fungicidal capacity of macrophages, through binding to mannose receptors and inducing IL-18 production [73, 165].

Particularly, a serine-thiol extracellular proteinase (PbST) with hydrolytic activity at 37°C has been reported in the pathogenic yeast phase of *P. brasiliensis* [166, 167], in line with its transcript upregulation [38]. This serine proteinase is involved in the cleavage of the main components of the basal membrane in vitro, including laminin, fibronectin, collagen type IV, and proteoglycans, suggesting a potential role for fungal tissue invasion and dissemination. In a *P. brasiliensis* vesicle proteome study, a subtilase-type proteinase psp3 (PADG_07422) was identified [59]. Its identity could be PbST, since it showed a free cysteine residue in its sequence; however, further experimental evidence is still required.

Further escape of *Paracoccidioides* spp. from the immune system is done by altering T-cell repertoire. Differentiation and maturation of T-cells occurs in the thymus, thus integrity of the thymic microenvironment is crucial for the maturation of thymocytes. Experimental data in a murine model of acute paracoccidioidomycosis shows that infection with *Paracoccidioides* yeast cells promotes thymus atrophy as a consequence of epithelial cell spatial disarrangement and increased gene expression of inflammatory mediators [168, 169]. These results suggest that a decreased differentiation of pathogen-specific T-cells leads to host immunosuppression, favoring *Paracoccidioides* spp. ability to thrive and multiply in the thymus microenvironment (Figure 2(c)).

## 6. Conclusions

Recent molecular evolutionary studies have shown differences in the ecoepidemiology of *Paracoccidioides* spp. [8, 170], suggesting diversifying mechanisms of pathogenicity and intracellular survival across these species that could also be explained by the complex and stochastic adaptation process of evolving within two particular ecological niches, the soil and live tissues of animal hosts. Likewise, Pigosso et al. [171] have demonstrated that the genus *Paracoccidioides* have important differences in their metabolic profiles, which must play a critical role during the host-pathogen interaction at the onset of the infection. However, while there is room for mammalian virulence adaptation in *Paracoccidioides* spp., it is important to always have in mind that virulence is a microbial property exclusively expressed in a susceptible host, and the outcome of this interaction is dependent on both players [172]. PCM is mostly related to low-income male workers on rural endemic areas of Central and South America, which are often related to rural poverty and malnutrition.

Over the last years, using antisense RNA technology, significant progress has been made to enhance our understanding of *Paracoccidioides* spp. host-pathogen interaction, pathogen resistance, and fungal virulence. In Table 4, we summarized genes shown by functional molecular studies using antisense technology to be involved in virulence and/or immune-evasion strategies from the host. In the near future, with the emergence of CRISPR technology and full access to diverse databanks (complete genomes, transcriptome, proteomic, metabolomics, lipidomics, etc.), we will gain more knowledge on the virulence processes that eventually should translate into patient's benefits.

## Figures and Tables

**Table 1:** Relative content of the main polysaccharides present in the yeast cell wall of different strains of *Paracoccidioides brasiliensis*. Strains belonging to at least three different cryptic species were grown at 37°C on RPMI 1640 (Gibco) liquid medium, buffered with 0.165 M morpholinepropanesulfonic acid (MOPS) to pH 7.0 for 4 days.

Morphological phase	Polysaccharide	Polysaccharide content per *P. brasiliensis* strain (cryptic species)				
		Pb73 (PS3)	Pb300 (PS4)	Pb377 (PS4)	Pb444 (PS4)	Pb381 (S1)
M	*α*-(1,3)-Glucan	1.06 ± 0.5	tr	tr	tr	7.0 ± 0.3
	*β*-(1,3)-Glucan	31.4 ± 0.4	25.4 ± 0.1	27.7 ± 0.2	20.2 ± 1.3	22.2 ± 1.1
	Chitin	13.2 ± 0.7	17.3 ± 0.4	12.6 ± 0.6	8.6 ± 0.2	13.5 ± 0.5

Y	*α*-(1,3)-Glucan	22.4 ± 0.9	23.7 ± 0.2	23.8 ± 0.4	24.1 ± 0.8	32.6 ± 1.0
	*β*-(1,3)-Glucan	10.6 ± 0.6	6.8 ± 0.5	3.9 ± 0.2	8.6 ± 0.4	6.3 ± 0.3
	Chitin	35.1 ± 1.3	31.4 ± 0.6	18.0 ± 0.2	26.6 ± 0.8	23.5 ± 0.8

tr stands for traces.

**Table 2:** Relative content of the main polysaccharides present in the yeast cell wall of *Paracoccidioides brasiliensis* strain Pb73 yeast cells, grown on different culture media for 4 days at 37°C. HS, horse serum.

Cell wall polysaccharide content	*P. brasiliensis* strain Pb73, yeast phase Grown on		
	RPMI	YPD	YPD + 5%HS
*α*-(1,3)-Glucan	22.4 ± 0.9	17.91 ± 0.17	32.52 ± 1.05
*β*-(1,3)-Glucan	10.6 ± 0.6	5.83 ± 0.28	5.14 ± 0.07
Chitin	35.1 ± 1.3	15.75 ± 0.27	12.87 ± 0.32

**Table 3:** Biochemical study of *P*. *brasiliensis* and *P. lutzii* cell wall composition in the presence of horse serum. Yeast cells were grown on YPD or YPD supplemented with 5% horse serum for 4 days at 37°C. HS, horse serum.

Cell wall polysaccharides content	*P. brasiliensis* strain Pb73, yeast phase Grown on		*P. lutzii* strain Pb01, yeast phase Grown on	
	YPD	YPD+ 5%HS	YPD	YPD + 5%HS
Chitin	15.7 ± 0.3	12.8 ± 0.3	21.1 ± 0.7	21.4 ± 1.0
*α*-(1,3)-Glucan	17.9 ± 0.2	32.5 ± 1.1	25.7 ± 0.4	25.2 ± 0.3
*β*-(1,3)-Glucan	5.8 ± 0.3	5.1 ± 0.1	3.8 ± 0.3	2.3 ± 0.3

**Table 4:** *Paracoccidioides* spp. genes shown by functional molecular studies using antisense technology to be involved in virulence and/or immune-evasion strategies from the host.

Gene	Encodes	Biological role	References
Pb*CDC42*	Rho GTPase	(i) Coordination of cell growth/morphogenesis of yeast cells, promoting an enhanced ability to evade the host immune system.	[45]

Pb*HAD32*	Hydrolase	(i) Adhesin involved in initial attachment of the infectious particles to the lungs.	[80, 81]

Pb*AOX*	Oxidase part of the electron transport chain in mitochondria	(i) Essential during the establishment of the fungal infection, possibly by assisting redox balancing during cell growth and the morphological switch.	[151, 152, 154]

Pb*HSP90*	Molecular chaperone	(i) Binds and stabilizes calcineurin thus controlling the cell differentiation.	[29–31]
		(ii) Essential upon thermo-dependent response and oxidative injury promoting fungal adaptation to the host.	

Pb*GP43*	Cell-surface component	(i) Adhesin that inhibits the phagocytic and fungicidal capacity of macrophages, through binding to mannose receptors and inducing IL-18 production.	[73, 76, 106, 107]
		(ii) Ability to reduce nitric oxide levels.	
		(iii) Adherence receptor in the internalization of the yeast into the host cell altering its cytoskeleton structure.	
		(iv) Modulation of host cells apoptosis.	

Pb*P27*	Protein mainly localized in cytoplasm and cell wall of yeast cells	(i) Involved in the yeast cellular morphological and glucose metabolism.	[173]
		(ii) Possible role in promoting latency in the host.	

Pb*Rbt5*	Surface glycosylphosphatidylinositol- (GPI-) anchored protein	(i) Hemoglobin uptake as an iron source for intracellular survival.	[158]
		(ii) Potential virulence factor.	

Pb*CCP*	Cytochrome c oxidase	(i) Avoids cell damage caused by nitrosative and oxidative stress.	[148, 150]
		(ii) Promote fungal survival within macrophages.	
		(iii) Potential virulence factor.	

Pb*SOD1*	Cytosolic superoxide dismutase	(i) Defense against endogenous-produced ROS.	[155]

Pb*SOD3*	Extracellular superoxide dismutase	(i) Pronounced extracellular activity involved in combating superoxide radicals generated during the host-pathogen interaction.	[155]
		(ii) Potential virulence factor.	

Pb*14-3-3*	30 kDa protein	(i) Adhesin able to bind laminin.	[105–107]
		(ii) Critical role in attachment/infection to host components and fungal virulence.	
		(iii) Involved in the morphological switching, ergosterol biosynthesis, and modulating apoptosis of host phagocytic and epithelial cells.	

Pb*SCONC*	Member of the Toll-like receptor family encoding a negative regulator of the inorganic sulfur assimilation pathway	(i) Dimorphism regulator by modulating the inorganic sulfur metabolism and influencing virulence.	[174]
		(ii) Novel virulence determinant.	

Pb*CATA*	Catalases	(i) Major role in endogenous ROS homeostasis in *Paracoccidioides* cells.	[156]
Pb*CATB*			

Pb*CATP*	Catalases	(i) Mainly triggered in the presence of exogenous ROS and highly relevant for fungal virulence.	[156]
